# Population Pharmacokinetics of Phosphocreatine and Its Metabolite Creatine in Children With Myocarditis

**DOI:** 10.3389/fphar.2020.574141

**Published:** 2020-11-16

**Authors:** Huan He, Meng Zhang, Li-bo Zhao, Ning Sun, Yi Zhang, Yue Yuan, Xiao-ling Wang

**Affiliations:** ^1^ Clinical Research Center, Beijing Children’s Hospital, Capital Medical University, National Center for Children’s Health, Beijing, China; ^2^ Department of Cardiology, Beijing Children’s Hospital, Capital Medical University, National Center for Children’s Health, Beijing, China

**Keywords:** pediatric myocarditis patients, phosphocreatine, creatine, population pharmacokinetics, simulation

## Abstract

**Objective:** This study aimed to develop a parent-metabolite joint population pharmacokinetic model to characterize the pharmacokinetic (PK) profile for phosphocreatine (PCr) and its metabolite creatine (Cr) in children with myocarditis and to use this model to study the PK profile of different dosing schemes.

**Methods:** One hundred pediatric patients with myocarditis were enrolled. Blood samples were collected at baseline and approximately 30, 40 or 50, 75, and 180 min after a single dose of phosphocreatine sodium. Plasma PCr and Cr concentrations were determined using an HPLC-MS/MS method. A nonlinear mixed effect model approach was used to build the population pharmacokinetic model. After validation, the model was used for simulations to evaluate the PK profile of different dosing schemes.

**Results:** A total of 997 plasma concentrations (498 for PCr and 499 for Cr) were included in the analysis. A four-compartment chain model (central and peripheral compartments for both PCr and Cr) with the first-order elimination adequately characterized the *in vivo* process of PCr and Cr. Allometric scaling based on bodyweight was applied to the PK parameters. The covariate analysis identified that the glomerular filtration rate (GFR) was strongly associated with Cr clearance. Bootstrapping and visual predictive checks suggested that a robust and reliable pharmacokinetic model was developed. The simulation results showed that PCr had no accumulation *in vivo*. With the infusion of PCr, the concentration of Cr increased rapidly.

**Conclusion:** A joint population pharmacokinetic model for PCr and Cr in pediatric patients with myocarditis was successfully developed for the first time.

## Introduction

Pediatric myocarditis is a common disease of the cardiovascular system in children (Bejiqi et al., 2019). It has become a serious threat to the life and health of children owing to its complex clinical manifestations, rapid illness development, and lack of specificity diagnosis methods (Niu et al., 2015).

Phosphocreatine (PCr) is an important endogenic high-energy phosphate compound in humans and mammals. It has a crucial role in cellular energy metabolism by acting as an immediate temporal and spatial energy buffer for intracellular energy transport (Gabr et al., 2011; Wallimann et al., 2011). PCr is metabolized into creatine (Cr) and generates ATP by creatine kinase (Neubauer, 2007). ATP consumption during myocardial injury is mainly replenished by PCr transformation. At present, PCr can be obtained by artificial synthesis. Exogenous complementary PCr is believed to help solve the most fundamental problem of energy metabolism in damaged myocardium, and it has been used as a cardioprotective drug and used in patient therapy after heart failure or myocardial infarction (Sharov et al., 1986; Saks et al., 1992; Kôrge et al., 1998; Strumia et al., 2012).

Exogenous PCr has also begun to be used in the therapy of pediatric myocarditis (Niu et al., 2015). In addition, it has also been recorded in the Chinese National Formulary (page 212, the chemical and biological products for children) and the Chinese Pharmacopoeia (2015) as the drug for viral myocarditis. The pharmacokinetics (PK) of PCr in adults and animals has already been studied (Afonskaia et al., 1985; Lorenzi et al., 1987; Xu et al., 2014). After intravenous PCr in mice, the changes in PCr concentrations were consistent with the two-compartment model, and most PCr is metabolized to Cr (Xu et al., 2014). The PK studies of PCr in adults also showed that the elimination of PCr in the human body was two-phase elimination (Lorenzi et al., 1987). However, the distribution of its major metabolite Cr in the human body has not been studied.

The currently available information about PCr in children is limited and is considered “off-label use.” The PK of PCr and Cr in children is unknown, and interindividual variability (IIV) is not clear.

To resolve the above problems, a study of the population pharmacokinetics of PCr and its metabolite Cr in Chinese children was performed at Beijing Children’s Hospital. This study was designed to understand the pharmacokinetic behavior of PCr and Cr in children after intravenous infusion and identify significant factors influencing PK parameters to provide evidence for rational use in children.

## Methods

### Subject Selection

This study was conducted at Beijing Children’s Hospital, Capital Medical University. Inclusion criteria were 1) age less than 18 years, 2) male or female, 3) clinically diagnosed pediatric myocarditis, and 4) the acute stage (new onset of myocarditis and symptoms were obvious and varied and generally, the course of the disease is less than half a year) in the clinical stage of myocarditis. The main exclusion criteria were as follows: allergy to phosphocreatine sodium, other causes of myocardial injury, a history of heart disease, other serious illnesses, other causes of electrocardiogram changes, participation in other clinical trials prior to screening, and renal insufficiency. The baseline information included gender, age (year), height (cm), body weight (BW, kg), disease duration, white blood cell count (WBC), red blood cell count (RBC), hemoglobin (HGB), blood platelet count (PLT), percentage of neutrophils (NEUT), lymphocyte percentage (LYMPH), percentage of monocytes (MONO), percentage of eosinophilic cells (EO), percentage of basophilic cells (BASO), total protein (TP), albumin (ALB), alkaline phosphatase (ALP), aspartate transaminase (AST), alanine transaminase (ALT), total bilirubin (TBIL), direct bilirubin (DBIL), urea, creatinine, uric acid (UA), creatine kinase (CK), creatine kinase-MB (CK-MB), lactic dehydrogenase (LDH), alpha-hydroxybutyric dehydrogenase (HBDH), and hypersensitive troponin-I (HSTN-I). The details of these clinical data characteristics are provided in Table 1. The glomerular filtration rate (GFR) for children older than 1 and younger than 18 was calculated using the creatinine-based “Bedside Schwartz” equation (Eq. (1), currently considered the best method for estimating GFR in children) (Schwartz et al., 2009). For children under 1 year, the GFR was estimated using the original Schwartz equation (Eq. (2)) (Schwartz et al., 1976). This clinical trial protocol was approved by the Ethics Committee of Beijing Children’s Hospital (Approval number: [2016]-Y-013-C-02). Written informed consent was obtained from each enrolled patient or their legally authorized guardian.

(1) GFR=0.413×height (cm)/serum creatinine (mg/dL)

(2) GFR=0.45×height (cm)/serum creatinine (mg/dL)

**TABLE 1:** The demographic characteristics and covariates of the study population.

Parameters	Median (min, max)
Age (year)	5.7808 (0.3753, 16.4521)
Height (cm)	115.0 (63, 180)
Bodyweight (kg)	20.4 (7.9, 86)
Disease duration (day)	10 (1, 150)
WBC (10^9^/L)	7.7 (3.72, 19.43)
RBC (10^12^/L)	4.75 (3.36, 5.7)
Hemoglobin (g/L)	131 (105, 170)
PLT (10^9^/L)	317.0 (144, 708)
NEUT (%)	46.35 (19.8, 80.6)
LYMPH (%)	43.95 (9.5, 69.2)
MONO (%)	5.85 (0.9, 18.8)
EO (%)	2.25 (0, 8.6)
BASO (%)	0.30 (0.1, 3.1)
TP (g/L)	68.3 (49.8, 81.3)
ALB (g/L)	42.85 (35, 49.2)
ALP (U/L)	238.5 (77, 557)
AST (U/L)	27.7 (11.9, 147.5)
ALT (U/L)	13.60 (6.8, 342.1)
TBIL (μmol/L)	8.07 (3.52, 25.71)
DBIL (μmol/L)	0.98 (0.11, 8.06)
Urea (mmol/L)	3.97 (1.36, 7.93)
Creatinine (μmol/L)	32.8 (16.2, 75.2)
UA (μmol/L)	262.0 (97, 745)
CK (U/L)	89.5 (17, 521)
CK-MB (U/L)	22.5 (10, 322)
LDH (U/L)	235.5 (136, 406)
HBDH (U/L)	199.0 (117, 358)
HSTN-I (ng/ml)	0 (0, 0.006)
GFR(ml/min/1.73 m^2^)	127.7805 (66.33, 224.01)

WBC, white blood cell count; RBC, red blood cell count; PLT, blood platelet count; NEUT, percentage of neutrophils; LYMPH, lymphocyte percentage; MONO, percentage of monocytes; EO, percentage of eosinophilic cells; BASO, percentage of basophilic cells; TP, total protein; ALB, albumin; ALP, alkaline phosphatase; AST, aspartate transaminase; ALT, alanine transaminase; TBIL, total bilirubin; DBIL, direct bilirubin; UA, uric acid; CK, creatine kinase; CK-MB, creatine kinase-MB; LDH, lactic dehydrogenase; HBDH, alpha-hydroxybutyric dehydrogenase; HSTN-I, hypersensitive troponin-I; GFR, glomerular filtration rate.

### Study Design

Participants received a single intravenous infusion of phosphocreatine sodium according to their age (28 days ∼ < 1year old: 0.5 g, 1 ∼ < 6 years old: 1 g, and 6 ∼ < 18 years old: 2 g). This dosing strategy was determined by the clinician according to their experience and local protocols. The above phosphocreatine sodium was dissolved in a 50 ml 0.9% sodium chloride injection and continuously injected with an intravenous pump within 30 ± 2 min. Blood samples from the arm without an intravenous infusion were collected at baseline (before infusion) and approximately 30, 40 or 50, 75, and 180 min after the start of infusion. The sampling time point captured the rapid elimination (30, 40, or 50 min) and the end of elimination (75 and 180 min), which provided comprehensive information according to preliminary experiments. The actual infusion time and sampling time were recorded. The blood samples were centrifuged at 4°C (3,000 rpm) for 10 min, and the plasma in the upper layer was separated and stored immediately at −40°C until analysis.

### Sample Assay

An improved HPLC-MS/MS method based on previous literature (Sun et al., 2019) was used to measure plasma concentrations of PCr and Cr using salidroside and creatine-(methly-d3) (Cr-d3) as internal standards, respectively. Fifty microliters of plasma and 10 μL of internal standard solution (20 μg/ml salidroside and 100 μg/ml Cr-d3 for PCr and Cr determination, respectively) were prepared by protein precipitation with acetonitrile/water (1,000 μL, 1:1, v/v). After vortexing and centrifugation, 100 μL of the supernatant fluid was diluted with 500 μL of solvent, and 10 μL was injected into the HPLC-MS/MS system for the determination of PCr. For Cr determination, 20 μL of the supernatant fluid was diluted with 1,000 μL of solvent, and 5 μL was injected into the HPLC-MS/MS system.

A Hypersil Gold C_18_ column (150 × 2.1 mm, 5 μm, Thermo Scientific, United States) was used for the separation of PCr. The mobile phase was solution A: 2 mM ammonium acetate in water (pH 10, adjusted with ammonia) and solution B: methanol. Chromatography separation was obtained with gradient elution solvent, and the gradient elution program was as follows: 0–1.5 min, 98–20% A; 1.5–4 min, 20% A; 4–4.01 min, 20–98% A; 4.01–10 min, 98% A. Monitored ion pairs were m/z 210 → 79 and m/z 299 → 119 for PCr and salidroside in negative ionization mode, respectively.

For the separation of Cr, a Hypersil Gold C_18_ column (150 × 4.6 mm, 5 μm, Thermo Scientific, United States) was used. The mobile phase was solution A: 2 mM ammonium acetate in water (0.4‰ formic acid) and solution B: acetonitrile. Isocratic elution with 30% A was obtained. Monitored ion pairs were m/z 132 → 90 and m/z 135 → 93 for Cr and Cr-d3 in positive ionization mode, respectively.

This method was accurate with intraday and interday precision less than 6%. The lower limits of quantification (LLOQs) for PCr and Cr were 1.96 μmol/L and 30.53 μmol/L, respectively. Measured concentrations below LLOQ were marked in the dataset. The LLOQ of this method is limited by endogenous background concentrations in the particular batches used as blanks in building the calibration curves, rather than by the analytical sensitivity (Thakare et al., 2016).

### Population Pharmacokinetic Model Development

A sequential two-step analysis approach to modeling building was implemented (Patel et al., 2017). First, a population pharmacokinetic model of PCr was developed, and then parent parameters were fixed to develop the population pharmacokinetic model for Cr. The nonlinear mixed effect modeling method was used to establish the population pharmacokinetic model. The PCr and Cr concentrations were fitted using Phoenix NLME software (version 8.2, Certara, St. Louis, MO) with the first-order conditional estimation-extended least squares (FOCE-ELS) method throughout the model building process. Pharmacokinetic data below the LLOQ were analyzed using the M3 method (Beal, 2001). Model selection criteria were based on goodness-of-fit plots, objective function value (OFV, equal to −2 log-likelihood), Akaike information criteria (AIC), and precision of parameter estimates. The complex parent-metabolite joint population pharmacokinetic model was established in a stepwise fashion. First, the base model including PCr and Cr was developed with a residual error model and interindividual variability model. Then, covariate effects on the base model were investigated to construct the covariate model.

For PCr, the structural model was tested using either one-compartment or two-compartment PK models. The one-compartment model parameters included the volume of distribution (V) and central clearance (CL). For the two-compartment structural model, intercompartmental clearance (Q) and volume of the second compartment (V2) were included. The PCr was an endogenous compound, and a parameter of baseline PCr was tested to incorporate in the model. However, the predose PCr levels were all below the LLOQ; therefore, the baseline PCr was assumed to be 0. The structural model for Cr was also tested. The fraction (F_m_) of PCr metabolized to Cr was fixed to avoid identifiability problems, and the volumes of the compartment for Cr were estimated. A published study reported that a large amount of PCr was metabolized as Cr in animals after intravenous injection of exogenous PCr, and the conversion rate was approximately three-quarters (Xu et al., 2014). In this study, according to the references and the characteristics of the drug concentration-time curves of PCr and Cr, F_m_ of PCr converted to Cr was assumed to be 0.75. Because of the existence of endogenous Cr, which was detected at each of the predose samples, a parameter of the baseline of endogenous Cr levels was added. Allometric scaling based on bodyweight (BW) was applied to the PK parameters. An allometric power model was used with power exponents fixed at 0.75 for clearances and 1.0 for volumes of distribution, and the maturation model was tested, as described in the following equations (Björkman et al., 2009; Hazendonk et al., 2016; Li et al., 2018):

(3) $${\text{CL}}_{\text{i}}={\text{θ}}_{\text{CL}}\times {\left({\text{BW}}_{\text{i}}/20\right)}^{0.75}\times \text{MF}$$

(4) $${\text{V}}_{\text{i}}={\text{θ}}_{\text{V}}\times {\left({\text{BW}}_{\text{i}}/20\right)}^{1}$$

(5) $$\text{MF}=\frac{1}{1+{\left(\text{age}/{\text{k}}_{50}\right)}^{\text{Hill}}}$$

In this expression, CL_i_ and V_i_ are the typical clearance and central volume of distribution for an individual i with bodyweight BW_i,_ while θ_CL_ and θ_V_ are the respective parameter values for a subject with a bodyweight of 20 kg. MF is the maturation factor, which is defined as the process of becoming mature. k_50_ is the age at which clearance maturation reaches 50% of that of adults, and Hill is the slope parameter.

The interindividual variability (η) of pharmacokinetic parameters was assumed to follow a log-normal distribution with a mean of 0 and a variance in ω^2^. An exponential formula was used to account for IIV (Eq. (6):

(6) $${\text{P}}_{\text{ij}}={\text{P}}_{\text{j}}\times {\text{e}}^{{\text{η}}_{\text{ij}}}$$

where P_j_ and P_ij_ represent the typical value of *j*th population parameter and *i*th individual’s *j*th parameter.

The proportional error model (Eq. (7), additive error model (Eq. (8), additive and proportional error model (Eq. (9)), and power error model (Eq. (10) were evaluated to describe the residual error:

(7) $${\text{C}}_{\text{ij}}={\text{IPRED}}_{\text{ij}}\times \left(1+{\text{ε}}_{\text{ij}}\right)$$

(8) $${\text{C}}_{\text{ij}}={\text{IPRED}}_{\text{ij}}+{\text{ε}}_{\text{ij}}$$

(9) $${\text{C}}_{\text{ij}}={\text{IPRED}}_{\text{ij}}\times \left(1+{\text{ε}}_{\text{ij},1}\right)+{\text{ε}}_{\text{ij},2}$$

(10) $${\text{C}}_{\text{ij}}={\text{IPRED}}_{\text{ij}}+{\text{IPRED}}_{\text{ij}}\times {\text{ε}}_{\text{ij}}$$

where C_ij_ is the observation concentration of the *i*th individual at the *j*th sampling point and IPRED_ij_ is the individual prediction value. The residual error (ε) is normally distributed with a mean of 0 and a variance in σ^2^.

The impact of covariates on pharmacokinetic parameters was evaluated. The relationships between covariates and IIV for pharmacokinetic parameters were investigated graphically ahead of covariate assessment. In this dataset, both categorical variables (gender) and continuous variables (height, BW, age, disease duration, WBC, RBC, hemoglobin, PLT, NEUT, LYMPH, MONO, EO, BASO, TP, ALB, ALP, AST, ALT, TBIL, DBIL, urea, creatinine, UA, CK, CK-MB, LDH, HBDH, HSTN-I, and GFR) were tested. Categorical covariates were included in the population model with the use of indicator variables, and the impact of the categorical covariates on each parameter was tested using a power function. Continuous covariates were centered at their median values, and the impacts of each covariate on parameters were evaluated using power functions. Stepwise forward addition and backward elimination were used to develop the covariate model. In the forward addition, a covariate was retained if it resulted in a reduction in the OFV > 6.64 (*p* < 0.01, df = 1). In the backward elimination, each covariate was left out of the full model built by the forward addition one at a time. A covariate was retained if the elimination of the covariate led to an increase in OFV > 10.83 (*p* < 0.001, df = 1).

### Goodness of Fit and Model Evaluation

To describe the adequacy of the final population pharmacokinetic model, goodness-of-fit plots including observations vs. population predictions, observations vs. individual predictions, conditional weighted residuals (CWRES) vs. population predictions, and CWRES vs. time were assessed. To evaluate the stability of the final pharmacokinetic model, a bootstrap resampling procedure was performed. A total of 1,000 bootstrap datasets were generated by random sampling with replacement, and the pharmacokinetic parameters were reestimated using the final population model. The median parameter value and their 95% confidence intervals (95% CIs) from bootstrap estimates were compared using the estimates of the final model. In addition, a visual predictive check (VPC) was used to assess the predictive ability of the final model. A total of 1,000 simulations of the final population pharmacokinetic model were performed. The VPC graphically showed the observations and different percentiles of simulated concentrations (5th, median, and 95th percentiles).

### Simulation

The final PCr and Cr joint population pharmacokinetic model was used to simulate different regimens for pediatric patients with myocarditis. Pediatric patients were divided into different subgroups according to age (Group 1: 28 days ∼ < 1 year old; Group 2: 1 ∼ < 6 years old; Group 3: 6 ∼ < 18 years old). Dose followed the dosage guidelines of this trial (Group 1: 0.5 g, Group 2: 1 g, Group 3: 2 g). Monte Carlo simulations were carried out to find the concentration curve under continuous drug administration. Based on the routine infusion time (30 min), multiple dosing simulations were performed to investigate the accumulation of PCr and Cr *in vivo* (regimens 1 and 2). Existing exogenous PCr is metabolized into creatine (Cr) and generates ATP by creatine kinase. ATP consumption during myocardial injury is mainly replenished by PCr transformation. However, PCr was eliminated quickly. We wanted to determine whether prolonged infusion time can improve the residence time of PCr *in vivo*. Therefore, 1,000 simulations were performed (regimens 3 and 4). The simulated regimens are as follows:

*Regimen 1*. The drug was intravenously administered once a day with an infusion time of 30 min for each infusion. The administration interval was 24 h, and the drug was administered continuously for 4 days.

*Regimen 2*. The drug was intravenously administered twice a day with an infusion time of 30 min for each infusion. The administration interval was 12 h, and the drug was administered continuously for 4 days.

*Regimen 3*. The drug was intravenously administered twice a day with an infusion time of 300 min for each infusion. The administration interval was 12 h, and the drug was administered continuously for 4 days.

*Regimen 4*. The drug was intravenously administered twice a day with an infusion time of 600 min for each infusion. The administration interval was 12 h, and the drug was administered continuously for 4 days.

## Results

### Patient Characteristics

**FIGURE 1:**
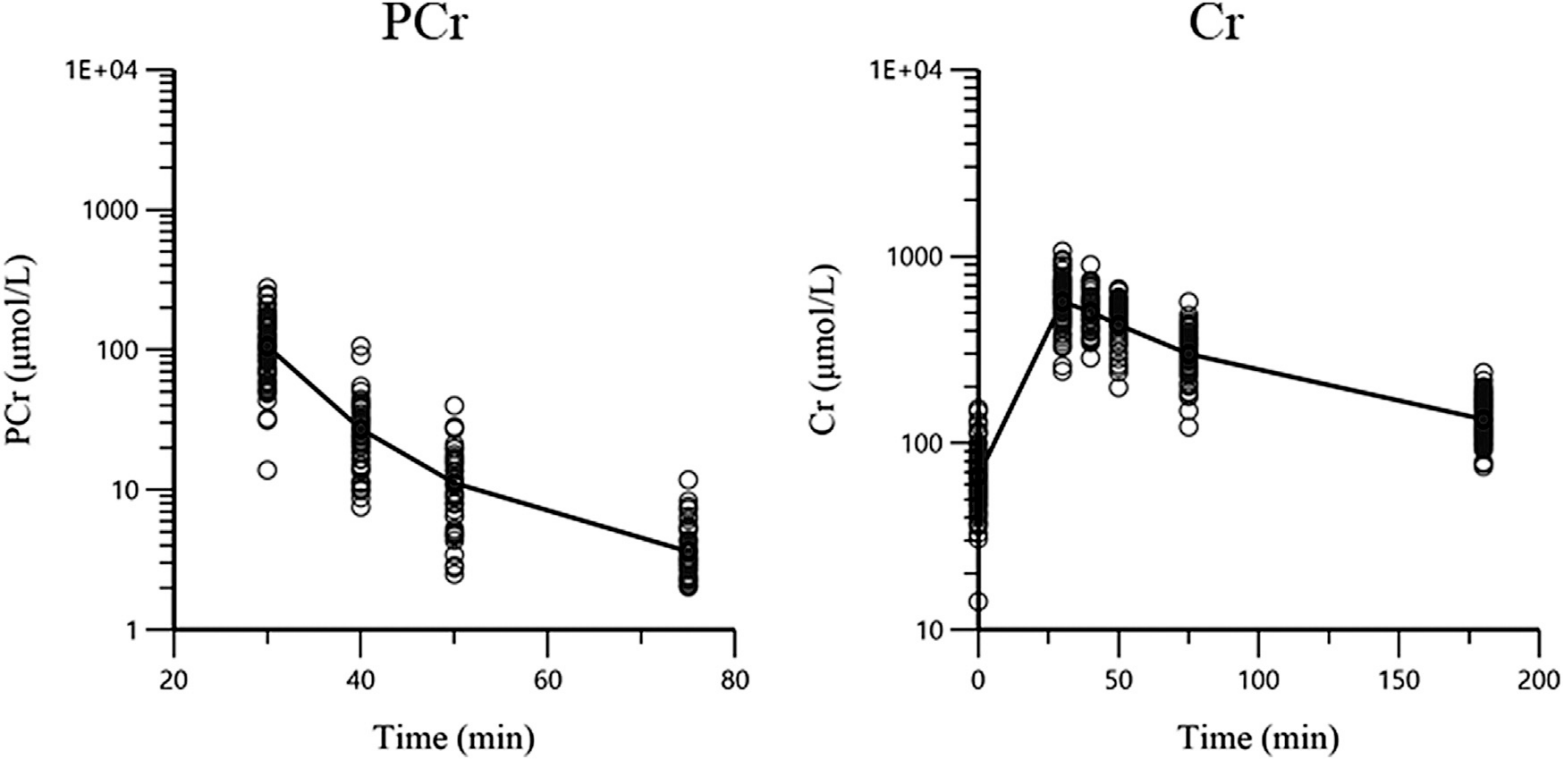
The concentration-time plots of PCr and Cr. The black lines are the mean concentration-time curves and circles are the observations.

A total of 100 pediatric patients (56 males and 44 females) with a median age of 5.78 years (range from 0.38 to 16.45 years) and a median BW of 20.4 kg (range from 7.9 to 86 kg) were enrolled in this study. Participant demographic characteristics are shown in Table 1. A total of 997 plasma concentrations (498 for PCr and 499 for Cr) were included in the analysis. Approximately 48.7% of the PCr concentrations were below the LLOQ and were analyzed using the M3 method (Beal, 2001). All Cr concentrations were above LLOQ. The ln concentration vs. time curve is shown in Figure 1. The baseline covariate information in this dataset is shown in Table 1.

### Population Pharmacokinetic Model

**FIGURE 2:**
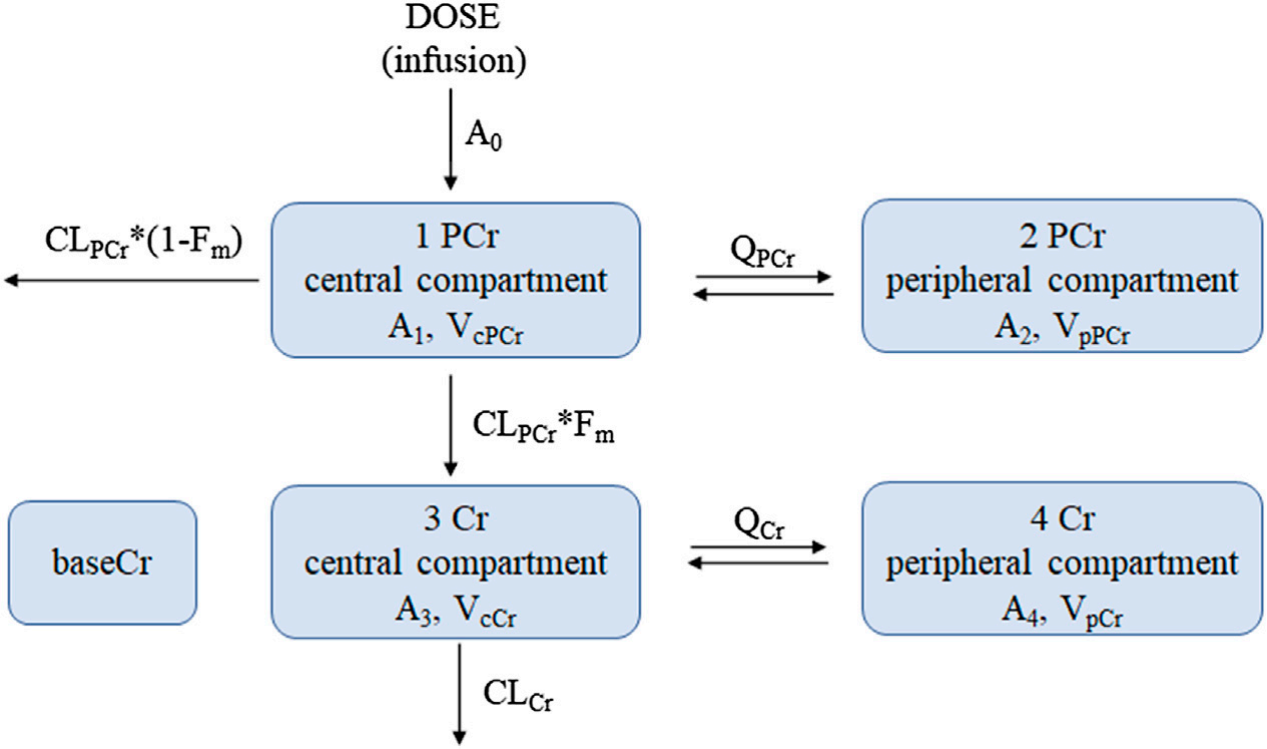
The proposed pharmacokinetic structural model of PCr and Cr in pediatric patients. **Equations (8)–(11)** were used to describe this model. Dose, dosage in the PCr central compartment (the zero-order administration rate A_0_); CL_PCr_, clearance of the PCr central compartment; Q_PCr_, intercompartmental clearance between the PCr central and peripheral compartments; V_cPCr_, distribution volume of the PCr central compartment; V_pPCr_, distribution volume of the PCr peripheral compartment; CL_Cr_, clearance of the Cr central compartment; Q_Cr_, intercompartmental clearance between the Cr central and peripheral compartments; V_cCr_, distribution volume of the Cr central compartment; V_pCr_, distribution volume of the Cr peripheral compartment; baseCr, the endogenous Cr concentration before drug administration. F_m_ is the fraction of PCr metabolized to Cr. A_1_ and A_2_ are the PCr amount in the central and peripheral compartments, respectively; A_3_ and A_4_ are the Cr amount in the central and peripheral compartments, respectively.

Based on the goodness of fit and the obtained OFV and AIC, a four-compartment chain model with the first-order elimination adequately described the PCr and Cr data. The schematic diagram is shown in Figure 2. The disposition of PCr in blood was characterized by a two-compartment model (compartments 1 and 2), which includes the central volume of distribution (V_cPCr_), the peripheral volume of distribution (V_pPCr_), the central clearance (CL_PCr_), and the intercompartmental clearance (Q_PCr_). The parent PCr was converted into metabolite Cr. The disposition of Cr in blood was described using another two-compartment model (compartments 3 and 4), including the central volume of distribution (V_cCr_), the peripheral volume of distribution (V_pCr_), the central clearance (CL_Cr_), and the intercompartmental clearance (Q_Cr_). The fraction (F_m_) of PCr metabolized to Cr was fixed to 0.75, and it was assumed that 75% PCr was eliminated via metabolic conversion to Cr. Another parameter of the baseline endogenic Cr (baseCr) was incorporated into the model to describe this basal value. The addition of the maturation model led to the model being unstable, and the estimates were unreasonable. Therefore, the maturation model was not appropriate. The following are the differential functions to describe the process:

(11) $$\frac{{\text{dA}}_{1}}{\text{dt}}={\text{A}}_{0}-\frac{{\text{CL}}_{\text{PCr}}}{{\text{V}}_{\text{cPCr}}}\times {\text{A}}_{1}+\frac{{\text{Q}}_{\text{PCr}}}{{\text{V}}_{\text{pPCr}}}\times {\text{A}}_{2}-\frac{{\text{Q}}_{\text{PCr}}}{{\text{V}}_{\text{cPCr}}}\times {\text{A}}_{1}$$

(12) $$\frac{{\text{dA}}_{2}}{\text{dt}}=-\frac{{\text{Q}}_{\text{PCr}}}{{\text{V}}_{\text{pPCr}}}\times {\text{A}}_{2}+\frac{{\text{Q}}_{\text{PCr}}}{{\text{V}}_{\text{cPCr}}}\times {\text{A}}_{1}$$

(13) $$\frac{{\text{dA}}_{3}}{\text{dt}}=\frac{{\text{CL}}_{\text{PCr}}}{{\text{V}}_{\text{cPCr}}}\times {\text{A}}_{1}\times {\text{F}}_{\text{m}}-\frac{{\text{CL}}_{\text{Cr}}}{{\text{V}}_{\text{cCr}}}\times {\text{A}}_{3}+\frac{{\text{Q}}_{\text{Cr}}}{{\text{V}}_{\text{pCr}}}\times {\text{A}}_{4}-\frac{{\text{Q}}_{\text{Cr}}}{{\text{V}}_{\text{cCr}}}\times {\text{A}}_{3}$$

(14) $$\frac{{\text{dA}}_{4}}{\text{dt}}=-\frac{{\text{Q}}_{\text{Cr}}}{{\text{V}}_{\text{pCr}}}\times {\text{A}}_{4}+\frac{{\text{Q}}_{\text{Cr}}}{{\text{V}}_{\text{cCr}}}\times {\text{A}}_{3}$$

(15) $$\text{CobsPCr}=\frac{{\text{A}}_{1}}{{\text{V}}_{\text{cPCr}}}$$

(16) $$\text{CobsCr}=\frac{{\text{A}}_{3}}{{\text{V}}_{\text{cCr}}}+\text{baseCr}$$

A_1_ and A_2_ are the PCr amounts in the central and peripheral compartments, respectively. A_3_ and A_4_ are the Cr amounts in the central and peripheral compartments, respectively. A_0_ represents the administration rate of creatine phosphate sodium in the blood. F_m_ represents the fraction of PCr metabolized to Cr. CobsPCr and CobsCr are the observed drug concentrations of PCr and Cr in the central compartment (plasma concentration), respectively. The definition of the other parameters (V_cPCr_, V_pPCr_, CL_PCr_, Q_PCr_, V_cCr_, V_pCr_, CL_Cr_, Q_Cr_, and baseCr) can be found in Figure 2.

The interindividual variability was estimated for CL_PCr_, V_cCr_, V_pCr_, CL_Cr_, and baseCr. The proportional error accounted for the residual error model of both PCr and Cr observations. Finally, the OFV of the base model was 6,785.535. According to the criteria of the forward inclusion and backward exclusion, covariate GFR was identified and included in the final population pharmacokinetic model. The incorporation of covariate markedly decreased the OFV from 6,785.535 to 6,773.0502 (△OFV = −12.4848). In the final model, GFR had a positive significant influence on CL_Cr,_ and their relationship was described by the following equation:

(17) $${\text{CL}}_{\text{Cr}}\;\left(\text{L/min}\right)=0.0825\times {\left(\text{BW/}20\right)}^{0.75}\times {\left(\text{GFR/}127.78\right)}^{0.311}\times {\text{e}}^{\eta {\text{CL}}_{\text{Cr}}}$$

In Eq. (17), 0.0825 L/min is the typical value of CL_Cr_, 20 kg is the median value of BW, 127.78 ml/min/1.73 m^2^ is the median value of GFR, and 0.311 is the correlation coefficient between BW and CL_Cr_. The estimated values of the parameters of the final model, their relative standard error (RSE), and interindividual variability are summarized in Table 2. All parameters were estimated with acceptable precision with RSE of estimates < 30%. The shrinkage in random IIV estimates for CL_PCr_, V_cCr_, V_pCr_, CL_Cr_, and baseCr was 3.93%, 10.3%, 22.6%, 5.22%, and 1.04%, respectively.

**TABLE 2:** Parameter estimates of the final population pharmacokinetic model and bootstrap results.

Final model parameter estimation				Bootstrap	
Parameter^a^	Estimate	RSE%	95% CI	Median	95% CI
V_cPCr_ (L)	8.22	10.1	6.59–9.86	8.12	5.82–9.60
V_pPCr_ (L)	3.07	10.9	2.42–3.73	3.25	2.76–6.35
CL_PCr_ (L/min)	1.33	3.09	1.25–1.41	1.32	1.25–1.40
Q_PCr_ (L/min)	0.136	30	0.0558–0.215	0.139	0.102–0.301
V_cCr_ (L)	2.39	8.17	2.01–2.77	2.43	2.04–2.82
V_pCr_ (L)	2.9	4.07	2.66–3.13	2.86	2.58–3.08
CL_Cr_ (L/min)	0.0825	1.64	0.0799–0.0852	0.0826	0.0799–0.0852
Q_Cr_ (L/min)	0.146	9.87	0.118–0.174	0.143	0.113–0.172
baseCr (μmol/L)	66.6	3.53	62.0–71.2	66.7	62.0–71.4
GFR on CL_Cr_	0.311	30	0.128–0.495	0.309	0.0874–0.528
Interindividual variability					
η _CLPCr_ (ω^2^)	0.0378 (CV% = 19.4)	14.2	0.0273–0.0483	0.0379	0.0263–0.0495
η _VcCr_ (ω^2^)	0.0882 (CV% = 29.7)	22.8	0.0488–0.128	0.086	0.0488–0.123
η _VpCr_ (ω^2^)	0.0354 (CV% = 18.8)	22.9	0.0195–0.0513	0.0360	0.0185–0.0535
η _CLCr_ (ω^2^)	0.0233 (CV% = 15.3)	18.2	0.0150–0.0316	0.0228	0.0148–0.0308
η _baseCr_ (ω^2^)	0.121 (CV% = 34.8)	21.8	0.0693–0.173	0.120	0.0685–0.172
Residual variability^b^					
σ _PCr_	0.244	6.79	0.212–0.277	0.243	0.213–0.273
σ _Cr_	0.0519	8.25	0.0435–0.0603	0.0510	0.0428–0.0600

CV, coefficient of variance; RSE, relative standard error.

a The typical values of parameters are presented for children with a bodyweight 20 kg and allometric power is fixed at 0.75 for clearances and 1.0 for volumes of distribution.

b The random residual model was described by the proportional error model.

### Goodness of Fit and Model Evaluation

**FIGURE 3:**
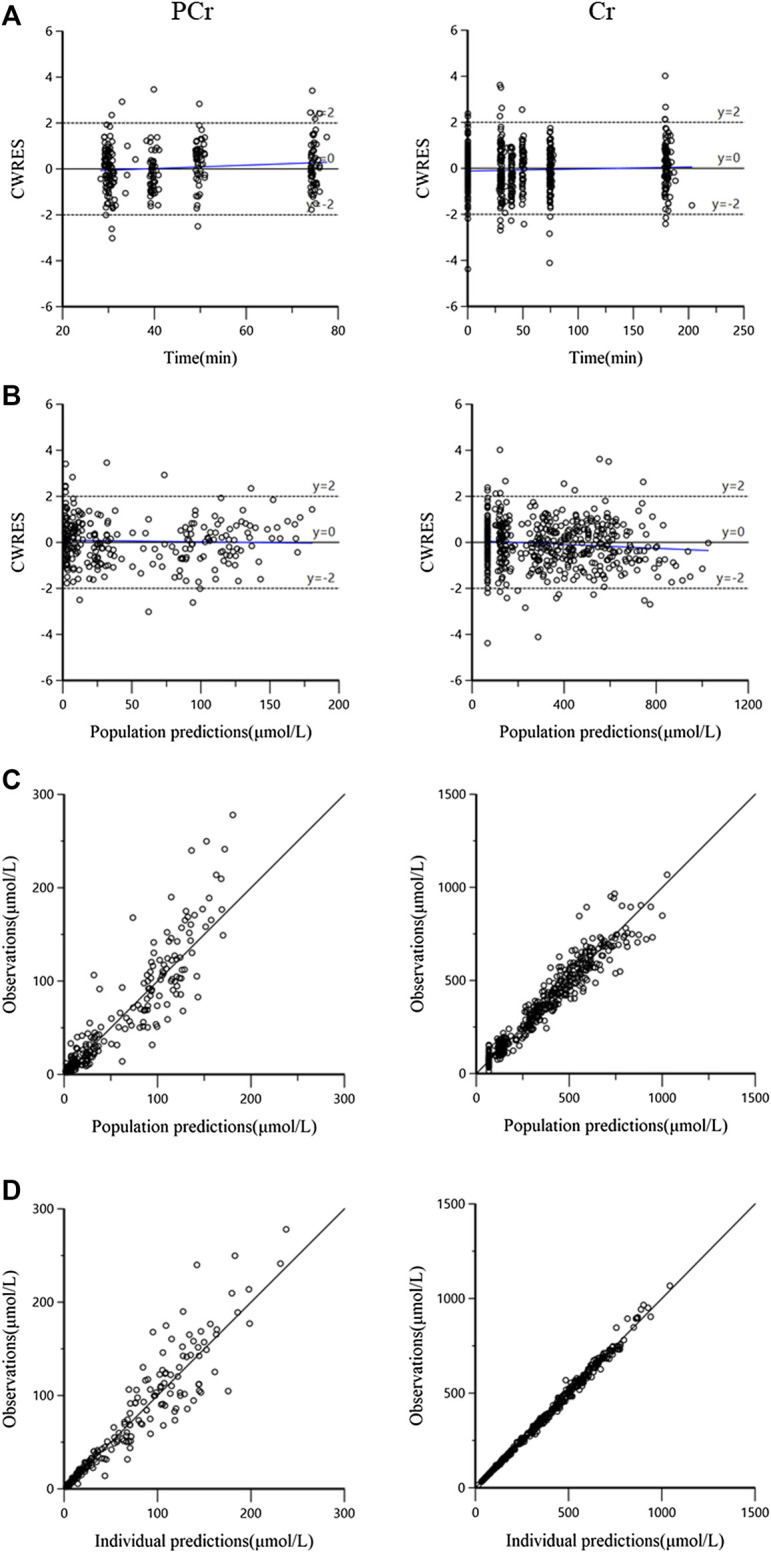
The goodness-of-fit plots for the final pharmacokinetic model. **(A)** Conditional weighted residuals (CWRES) vs. time, the blue solid line is the linear regression trend line; **(B)** CWRES vs. population predictions, the blue solid line is the linear regression trend line; **(C)** observations vs. population predictions, the black solid line means the line of unity y = x; and **(D)** observations against individual predictions, the black solid line is the line of unity y = x.

The goodness-of-fit plots for both PCr and Cr observations of the final model are shown in Figure 3. The plots for CWRES (PCr and Cr) vs. time or population predictions are shown in Figures 3A,B, which were used to detect any misspecifications in the model. The CWRES of the final pharmacokinetic model was distributed around the line y = 0, indicating that no apparent systematic bias was observed. Scatter plots of observation (PCr and Cr concentrations) vs. population or individual predictions are shown in Figures 3C,D, and the solid lines are the unity. The data points were distributed evenly around the line of identity in the final model (Figure 3C). These diagnostic plots suggested a good fit for the proposed final model to the data.

**FIGURE 4:**
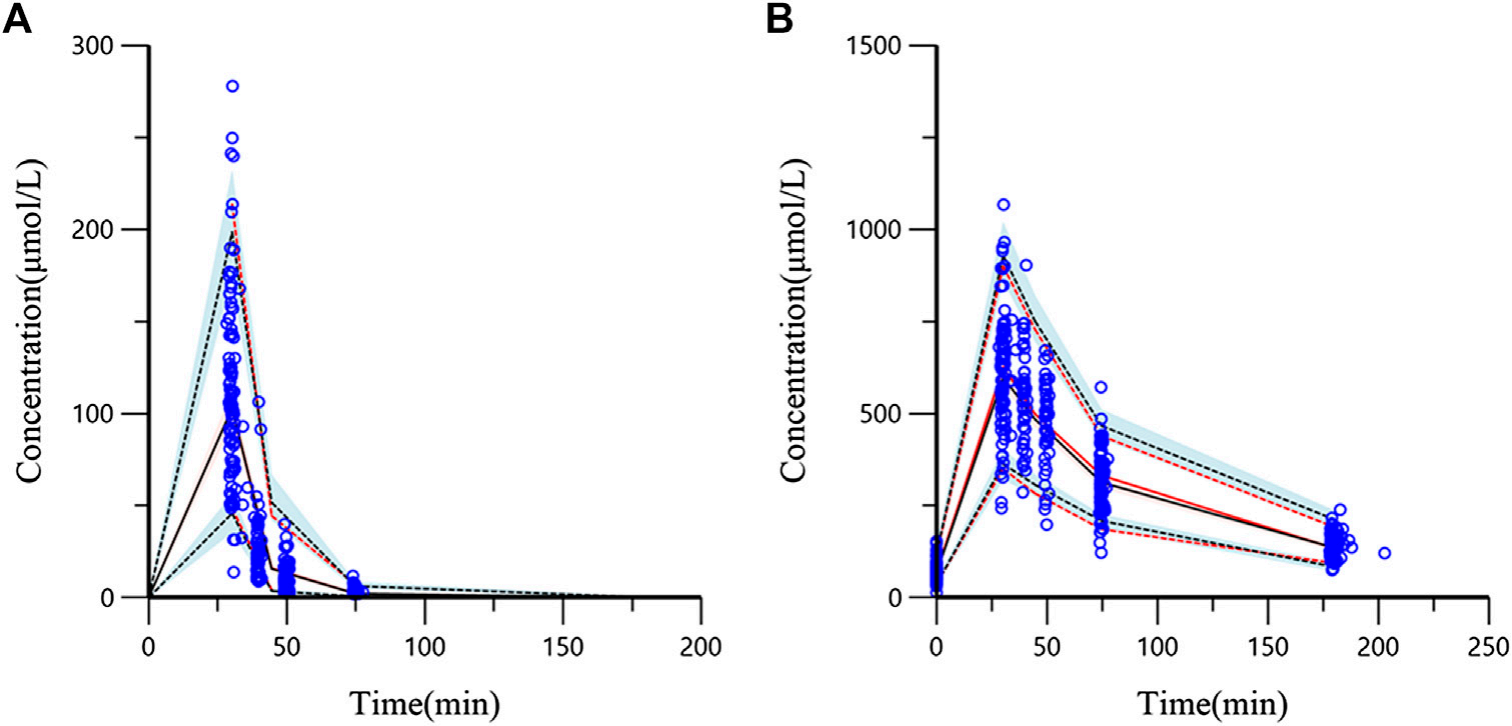
Visual predictive check (VPC) from the final population pharmacokinetic model (**(A)** PCr concentration; **(B)** Cr concentration). The blue circles are the actual original observations. The red solid line is the observed 50th percentile and the red dashed lines mean the 5th and 95th percentiles of observations. The black solid line means predicted 50th percentile and the black dashed lines represent the 5th and 95th percentiles from the simulated observations (shadow means 95% confidence band).

The evaluation of 1,000 bootstrap runs with a success rate of 98.3% (983 out of 1,000 resampling datasets were successful in optimization) showed an acceptable stability of the final population pharmacokinetic model. Bootstrap results of median parameter values and 95% CIs are listed in Table 2. The median values are similar to the final parameter estimates, and 95% CIs are close to the values obtained during the final data fitting. The VPC results (PCr and Cr) are shown in Figure 4. In the VPC plot, the 90% prediction interval (90% PI) is the area between the predicted 5th and 95th percentiles (black dashed lines), and the majority of actual observations fell within the 90% PI. The predicted 5th, 50th, and 95th percentiles (black lines) are closed with the observed 5th, 50th, and 95th percentiles (red lines), respectively. This plot indicated an adequate predictive ability of the final population model.

### Simulation

**FIGURE 5:**
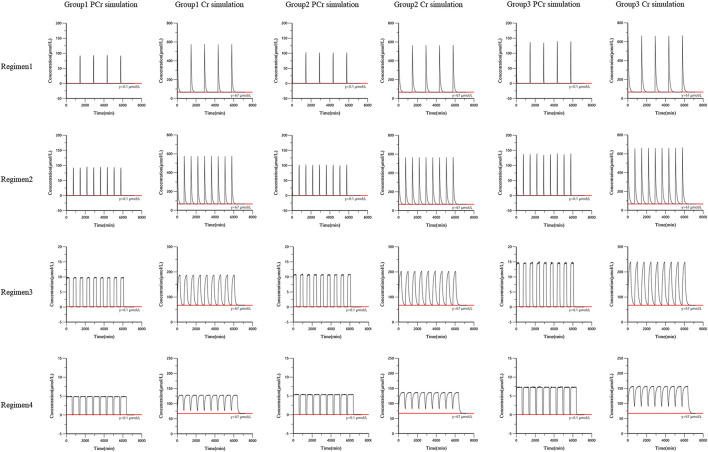
The simulation curves from the final population pharmacokinetic model. Black solid line was the 50th percentile of simulated concentrations. Red line was the reference line.

The simulation results showed that PCr had no accumulation *in vivo* based on the three simulation doses. Under regimens 1 and 2, PCr was basically eliminated within 3 h (the concentration of the 50th quantile of 1,000 simulations was less than 0.1 μmol/L). Increasing infusion time (regimens 3 and 4) could prolong the residence time of PCr *in vivo* and maintain a certain concentration of PCr. With the infusion of PCr, the concentration of Cr *in vivo* increased rapidly. In regimens 1 and 2, Cr was basically eliminated to the baseline level after 12 h (the concentration of the 50th quantile in 1,000 simulated profiles was similar to the baseline level 67 μmol/L). The simulation curves are shown in Figure 5.

## Discussion

Although the pharmacokinetics of PCr has already been investigated in adults, its pharmacokinetic characteristics in children are unclear, and its metabolite Cr was ignored. To the best of our knowledge, this is not only the first PK study of PCr in children but also the first comprehensive population pharmacokinetic model of PCr and its metabolite Cr. In this study, the pharmacokinetic characteristics of PCr and Cr in children were studied by a population pharmacokinetic method, and the established joint model fitted the concentration data well. This study could be helpful to further understand the PCr and Cr PK characteristics in children and provide a reference for pediatric clinical applications.

In the base model development process, the structural models of both PCr and Cr were tested with a one-compartment model and a two-compartment model, respectively. The PCr data were best fitted by a two-compartment model, which is in accordance with a previous report (Lorenzi et al., 1987; Xu et al., 2014). The structural model of Cr was best described by a two-compartment model with an estimation of the baseline level of Cr level (baseCr).

There was only a PCr dose for each subject, and Cr was generated by PCr metabolism. The distribution volume of Cr and F_m_ of PCr metabolized to Cr could not be estimated at the same time. It has been reported that a large amount of PCr was metabolized to Cr in animals after intravenous administration of PCr, and the conversion rate was approximately three-quarters (Xu et al., 2014). In the concentration-time plots (Figure 1), Cr concentration increased rapidly after PCr infusion. In our study, we were more interested in knowing the distribution of Cr in children. Therefore, the apparent distribution volume of Cr was estimated, and the fraction of PCr metabolized to Cr was fixed to avoid identifiability problems in the model building. There is no evidence that showed the exact ratio fraction of PCr to Cr in humans. The Cr concentration increased rapidly with the infusion of PCr seen in the concentration profiles, and a general view is that a large amount of PCr is metabolized to Cr to provide ATP after PCr injection (Neubauer, 2007). In this study, based on the results of animal experiments from the reference (Xu et al., 2014), F_m_ of PCr converted to Cr was assumed to be 0.75. This setting could ensure the estimation of the PK parameters of Cr in children and did not affect the estimation of PCr parameters. To compare the estimation results of different fraction assumptions, we developed a model based on the fraction fixed 1 or 0.5, and the estimation results showed that the PCr parameters did not change much.

BW was incorporated in the base model with an allometric exponent of 0.75 for the clearance parameters and 1 for the volume terms, which resulted in a large decrease in OFV. The addition of allometric scaling made the base model more stable and accelerated the speed of modeling. In this study, to identify as many factors that affect the *in vivo* behavior of PCr and Cr as possible, a large amount of information from the study sample was collected as covariates. All the covariates were introduced to the base model to go through a stepwise process. After forward inclusion and backward elimination, only GFR was identified as a key covariate for the clearance of Cr. The influence of GFR on CL_Cr_ was estimated with an exponent of 0.311, which means that a high level of GFR leads to an increase in Cr clearance. As GFR increased by 100%, Cr clearance increased by 24%.

The final PCr and Cr models were evaluated by the bootstrap and VPC methods, respectively. The results showed that the established model had good stability and predictability. The parameters estimated by the final model showed that the distribution volume of the PCr central compartment (V_cPCr_) was 8.22 L, the distribution volume of the PCr peripheral compartment (V_pPCr_) was 3.07 L, the total distribution volume was 11.29 L, and the clearance was 1.33 L/min in children who were 20 kg. After conversion, the apparent volume of distribution in adults who were 70 kg is approximately 39.5 L, and the clearance is approximately 3.40 L/min. It has been shown that the plasma concentration of PCr in adult healthy volunteers decreased in two phases after intravenous injection of 1 g phosphocreatine sodium. The apparent volume of distribution is approximately 50.3 L, and the clearance is approximately 1.627 L/min. Compared with the pharmacokinetic parameters in adult healthy volunteers, the PCr pharmacokinetic parameters obtained in this study had a slightly smaller distribution volume and a slightly higher clearance in children.

The distribution volume of the Cr central compartment (V_cCr_) was 2.39 L, the distribution volume of the Cr peripheral compartment (V_pCr_) was 2.9 L, and the clearance was 0.0825 L/min in children who were 20 kg. V_pCr_ is larger than V_cCr_, suggesting that Cr can be widely distributed in muscle and other tissues. This study first reported the distribution characteristics of Cr in children under F_m_ set to 0.75. The typical value of Cr base value (baseCr) is 66.6 μmol/L, which is approximately 8.73 mg/L, which is consistent with the normal content in the human body (7–13 mg/L) (Persky et al., 2003). The evaluated model was applied to simulate different regimens for subgroups with different ages. The simulation results suggested that PCr did not accumulate *in vivo*.

## Conclusion

In conclusion, the joint population pharmacokinetic model for PCr and its metabolite Cr in pediatric patients was successfully developed for the first time. The model adequately described the pharmacokinetics of both the parent drug and metabolite with individual predicted values close to the measured value. This model may be helpful for the pediatric clinical application of phosphocreatine.

## Data Availability Statement

The datasets presented in this article are not readily available because of privacy and ethical restrictions. Requests to access the datasets should be directed to X-LW, wangxiaoling@bch.com.cn.

## Ethics Statement

The studies involving human participants were reviewed and approved by Beijing Children’s Hospital, Capital Medical University. Written informed consent to participate in this study was provided by the participants' legal guardian/next of kin.

## Author Contributions

All authors contributed to the design and implementation of the study. L-BZ, YY, and X-LW designed the study. NS and HH acquired the data. MZ, YZ, and HH analyzed the data and develop the pharmacokinetic model. HH wrote the manuscript and L-BZ, YY, and X-LW revised it.

## Funding

This work was supported in part by grants from the National Science and Technology Major Project for Major New Drugs Innovation and Development (2017ZX09304029 and 2018ZX09721003).

## Conflict of Interest

The authors declare that the research was conducted in the absence of any commercial or financial relationships that could be construed as a potential conflict of interest.
